# Therapeutic efficacy of nutritional support by percutaneous endoscopic gastrostomy in critically ill patients: A self-control clinical trial

**DOI:** 10.12669/pjms.331.11627

**Published:** 2017

**Authors:** Fei Zhou, Ya-Ling Gao, Zheng-Jin Liu, Yi-Qun Hu

**Affiliations:** 1 Fei Zhou Departments of Gastroenterology, Zhongshan Hospital Affiliated to Xiamen University, Xiamen 361004, Fujian, China; 2 Ya-Ling Gao Departments of Gastroenterology, Zhongshan Hospital Affiliated to Xiamen University, Xiamen 361004, Fujian, China; 3 Zheng-Jin Liu Departments of Pathology, Zhongshan Hospital Affiliated to Xiamen University, Xiamen 361004, Fujian, China; 4 Yi-Qun Hu Departments of Gastroenterology, Zhongshan Hospital Affiliated to Xiamen University, Xiamen 361004, Fujian, China

**Keywords:** Critically ill patients, Nutritional support, percutaneous endoscopic gastrostomy

## Abstract

**Background & Objective:**

Percutaneous endoscopic gastrostomy (PEG) is a procedure to provide enteral nutrition for critically ill patients. It is commonly used in clinical practice; however, the widespread use of PEG is controversial. Our objective was to evaluate the therapeutic effect of nutritional support by PEG in these critically ill patients.

**Methods:**

A total of 64 critically ill patients including 41 males and 23 females (aged 23-84) were identified by the Acute Physiology and Chronic Health Evaluation (APACHE) II scoring system during September 2004 to June 2012. The nutritional status before and after PEG was mainly assessed by the tricep skinfold thickness and serum albumin level. The nutritional status and pathological condition were assessed at 4, 8 and 12 weeks before and after PEG feeding. The assessment was according to the classical method of the human nutritional status. Follow-up was performed at one month, three months and 1.5 year after gastrostomy. Statistical analysis was performed by SPSS 11.5 software. The incidence of inhalation pneumonia and gastroesophageal regurgitation was compared by chi square (χ^2^) test. P<0.05 were considered statistically significant.

**Results:**

Among the 64 patients, 9 patients died of their former diseases or related symptoms. Postoperative follow-up showed that both nutritional status and complications were improved after PEG in 55 patients (P<0.05). The serum albumin and tricep skinfold thickness levels were significantly increased. The incidence of hypoglycemia, hypocalcemia, hypokalemia and hyponatremia were lower than pre-operation. The frequencies of complications were significantly reduced. No severe complications occurred in any patient.

**Conclusions:**

Our study confirmed that PEG was a good long-term route of nutritional supply with no serious complications for critically ill patients.

## INTRODUCTION

Numerous conditions may compromise the passage of food along the digestive tract, such as severe head injury, brain tumors, cranial encephalic trauma, cerebral hemorrhage and cerebral infarction.^1^ Because of the impairment or dysfunction of gag reflex; they are unable to ingest food orally. For these critically ill patients, enteral nutrition support is an important part. Generally, for such patients, nasogastric tube (NG) feeding and intravenous nutrition are the traditional ways to provide nutrition support in the short-term. However, NG tube was often poorly tolerated by the patient, besides, its prolonged use may lead to some complications, including chronic sinusitis, lesions to the nasal wing, esophagitis and aspiration pneumonia.^2^,^3^ Additionally, the high cost of intravenous nutrition is a heavy burden for the patients. Therefore, for longer term enteral tube feeding, feeding via a gastrostomy tube is currently recommended.^4^ Since 1876, Verneuil has carried out gastrostomy successfully for long-term enteral feeding in patients with swallowing limitations. In 1980, Gauderer et al.^5^ invented a new technique of feeding tube placement in gastrostomy using endoscopy, namely percutaneous endoscopic gastrostomy (PEG). Presently, PEG is one of the most commonly performed gastrointestinal procedures with less serious complication.^6^-^10^

PEG is an endoscope-guided procedure to provide enteral nutrition support.^5^ It is applicable to several diseases, such as Parkinson, stroke sequela, and head and neck neoplasm.^7^ It has also been widely used in patients with laryngo-pharyngeal tumors who need chemotherapy or radiotherapy, as well as patients combined with severe respiratory diseases gastroesophageal regurgitation. It has been reported that PEG technique can largely reduce the major complications, patient discomfort and cost.^11^ Importantly, some studies have revealed that feeding via a PEG tube is superior to NG tube from both a clinical and nutritional perspective.^12^,^13^ Research has shown that PEG can significantly increase survival rate, reduce aspiration rate and the slippage of catheter.^14^ Therefore, PEG has been widely used as the substitution of NG tube in recent years. However, some patients still refuse to use PEG due to the risk of complications.^15^

In the present study, 64 critically ill patients were administered PEG. The preoperative and postoperative incidence of inhalation pneumonia and gastroesophageal regurgitation were compared. The aim of this study was to evaluate the therapeutic effect of nutritional support by PEG in these critically ill patients. We expected to provide valuable information on PEG in nutritional support for critically ill patients.

## METHODS

During September 2004 to June 2012, 64 critically ill patients including 41 males and 23 females identified by the Acute Physiology and Chronic Health Evaluation (APACHE) scoring system^16^ (APACHE II score ≥10, mean APACHE II score 17.8) were included in this study. Their age ranged from 23 to 84 (mean age 51.3). All patients had difficulties in swallowing, among whom, 17 were diagnosed with cerebral hemorrhage, 11 with cerebral infarction, 29 with head injury, and four with respiratory failure. In addition, two patients were prescribed chemotherapy for nasopharyngeal carcinoma and one was drowning resuscitation. Most patients had received NG or intravenous hyperalimentation therapy. Specially, the patients with surgical contraindications, including massive ascites, peritoneal dialysis and gastric varices, had been excluded.

Before the procedure, informed consents were signed by all patients or their first-degree relatives. This study was conducted in accordance with the declaration of Helsinki with approval from the Ethics Committee of Zhongshan Hospital Affiliated to Xiamen University Ethics Committee. Written informed consent was obtained from all participants.

### Preoperative preparation

Before PEG, all the subjects received nothing by mouth for more than 12 hours. Besides, they underwent routine electrocardiogram (ECG) monitoring and oxygen inhalation. Some patients also had endotracheal intubation. For the conscious patients, intravenous anesthesia with propofol was applied during the procedures.

### Surgical procedures

**Fig. 1:**
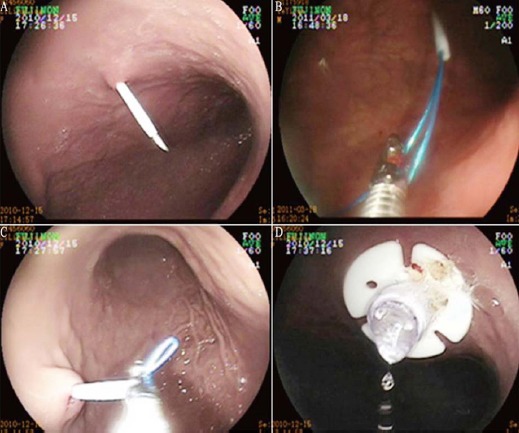
Percutaneous endoscopic gastrostomy (PEG) diagram. (A) Punctured and inserted the trocar; (B) introduced the guidewire out of the mouth cavity; (C) the PEG tube was introduced into the stomach; (D) fixed the PEG tube.

The patient was laid in a flat supine position. A local anesthetic was then infiltrated into the area around the puncture site the PEG tube was led into the stomach. (Fig.1).

### Postoperative care

After PEG, the patient received nothing by mouth for another 24 hours. Antibiotic (cefuroxime, 0.75 g, q8h or ceftriaxone, 1.0g, qd) was administered by intravenous dripping for three days for prophylaxis of infection. Elemental diet was given via the PEG tube after irrigating the tube with normal saline or lukewarm water 24 hours postoperatively.

### Assessment of nutritional indexes and pathological condition

The nutritional status and pathological condition were assessed at 4, 8 and 12 weeks before and after PEG feeding. The assessment was according to the classical method of the human nutritional status. The triceps skinfold thickness and serum albumin level were measured. In addition, the blood sugar, blood calcium, serum potassium and serum sodium were detected to compare with that of pre-operation. The body mass index (BMI) was not obtained because it was difficult to measure the body weight and height of these critically ill patients. The pathological condition of the critically ill patients was assessed by the APACHE II scoring system.

### Diagnosis and treatment of inhalation pneumonia and gastroesophageal regurgitation

Patients with difficulty in swallowing and regurgitation of food often had saliva, cough, followed by cough, with sputum and fever, which indicated that they may be suffering from inhalation pneumonia. Inhalation pneumonia was diagnosed by laboratory tests: white blood cells count and chest X-ray. The white blood cells count in patients with inhalation pneumonia was increased and the chest X-ray showed lung infection. Patients with pain of chest, heartburn, acid reflux, hiccups and reflux, such as recurrent vomiting and gastroesophageal reflux to the mouth, indicated gastroesophageal regurgitation.

### Follow-up

As most of the patients were critically ill, elderly or walking difficultly, the routine ways of follow-up including telephone follow-up and outpatient follow-up were chosen for patients. Follow-up was performed at one month, three months and 1.5 year after gastrostomy. Patients who were still in hospital were assessed directly (outpatient follow-up).

### Statistical analysis

Statistical analysis was performed by SPSS11.5 software (SPSS Inc., Chicago, IL, USA). The triceps skinfold thickness, serum albumin level and APACHE II score were compared by paired-t test before and after PEG. The incidence of inhalation pneumonia and gastroesophageal regurgitation was compared by chi square (χ^2^) test. P<0.05 were considered statistically significant.

## RESULTS

### General status of PEG

The operative time ranged from 15 to 20 minutes, and all vital signs were stable during the procedures of PEG placement. The nutritional support by intravenous pathway or NG was discontinued after PEG. Among the 64 patients, 9 patients died of their former diseases or related symptoms, such as head trauma, intracranial hemorrhage or malignant tumors; three patients were lost to follow-up after recovery. The rest 52 patients received a 1.5 year follow-up. Among these patients, the longest indwelling time of PEG tube was 14 months and the tube was replaced because of aging. The average PEG tube feeding was 6 months.

### Complications of PEG

During the follow-up period, complications occurred in some patients: one patient developed bacteremia; three patients had low-grade fever; four patients had skin infection; four patients had gastroesophageal regurgitation. The occurrence times of inhalation pneumonia reduced significantly (from 41 to 8) (P<0.01), and the incidence rate reduced from 64.1% to 13.3% (χ^2^=33.3). The incidence rate of gastroesophageal regurgitation reduced from 34.4% to 15% (χ^2^=6.2) (Table-I).

**Table-I:** Change of complications before and after percutaneous endoscopic gastrostomy (PEG).

Index	Before PEG	fter PEG	χ^2^ values
Inhalation pneumonia	41/64	8/60**	χ^2^=33.3
GER	22/64	9/60*	χ^2^= 6.2

Data were analyzed by χ^2^-test of SPSS 11.5.

* *P*<0.05 vs. data before PEG;

** *P* < 0.01 vs. data before PEG; GER: gastroesophageal regurgitation.

Intravenous antibiotic was used for patients with skin infection. For patients with gastroesophageal regurgitation, we used prokinetic agents, or increased the amount of the nutritional fluid. No digestive tract hemorrhage, perforation, pneumoperitoneum, necrotizing fasciitis, or any other severe complications occurred in any patient.

### Nutritional status

**Fig. 2:**
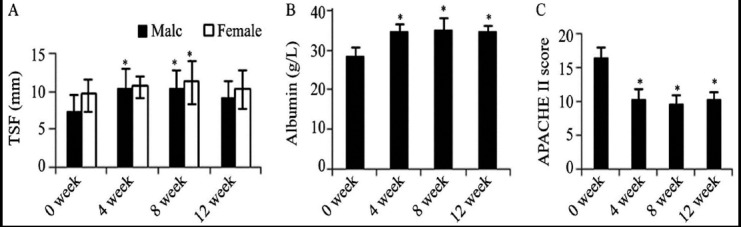
The (A) tricep skinfold thickness, (B) serum albumin levels (C) APACHE II score after percutaneous endoscopic gastrostomy (PEG). All data were shown as mean ± standard deviation. TSF: tricep skinfold thickness. * P<0.05 vs. 0 week.

The tricep skinfold thickness and serum albumin levels were significantly increased at 4^th^, 8^th^, 12^th^ week after PEG (P<0.05) (Fig. 2A, Fig. 2B), which indicated that the nutritional status was improved significantly. Except for 9 patients who died of the underlying critical conditions, symptoms of nausea and vomit, abdominal distension, and other presenting signs of malnutrition were improved; water-electrolyte imbalance and negative nitrogen balance were corrected. Additionally, the percentage of patients with low blood pressure, tachypnea or tachycardia reduced from 43.75% to 14.06%; the percentage of patients with arrhythmia decreased as well (26.56% vs. 12.50%); the incidence of hyponatremia, hypocalcemia and hypokalemia decreased by 61.25% (81.25% vs. 20.00%); the frequency of transient hypoglycemia was lower than preoperation (43.75% vs. 5.00%). As shown in Figure 2C, the APACHE II score decreased significantly at 4^th^, 8^th^, 12^th^ week after PEG (P<0.05), indicating that the pathological condition of the critically ill patients had improved as well.

## DISCUSSION

Critically ill patients are a group of patients with serious clinical symptoms that gravely influence patient normal life, activities and diet. The nutritional support of these patients has emerged as a vital component in the management of critically ill patients, because nutrition can supply vital cell substrates, antioxidants, vitamins, and minerals which are necessary for their recovery from illness.^17^ There are several kinds of methods for nutritional support, like intravenous nutritional, NG tube, laparotomic gastrostomy and PEG. In recent decades, PEG has been increasingly utilized as a option for nutritional support among patients who require long-term non-oral enteral feeding.^18^,^19^

Previous study has found that PEG feeding in malnourished patients with cystic fibrosis can improve nutritional status, and stabilize lung function, besides, it is suggested to be superior to using nasogastric tubes.^20^ In accordance with the findings above, our study indicated that PEG improved the nutritional status of the critically ill patients, manifesting as the increase of tricep skinfold thickness (Fig.2A) and serum albumin level (Fig.2B). Additionally, we also found that PEG significantly reduced the occurrences of common complications, including inhalation pneumonia and reflux esophagitis, which was consistent with the results reported before.^21^,^22^ Furthermore, APACHE II score was significantly reduced at 4^th^, 8^th^, 12^th^ week after PEG (P<0.05) (Fig.2C). However, PEG is not suitable for critical ill patients with severe underlying diseases or a relatively short expected life.^1^ For patients with pharyngeal and esophageal malignant tumors, PEG may run the risk of seeding cancer cells into the abdominal wall via the catheter. As shown by a catheter exfoliative brush cytology test, the occurrence rate of cancer cell seeding is about 27%.^23^ Therefore, case screening is extremely important.^24^ For such patients, comprehensive preoperative assessment is necessary.^25^ Although a large meta-analysis has reported a procedure-related morbidity of 9.4% and mortality of 0.53%,^26^ no patient died of PEG in our study. Thus, PEG can be regarded as a safe and effective method for nutritional support in critically ill patients.

PEG really brings some slight complications, such as, skin infection, gastroesophageal regurgitation, PEG tube displacement and catheter obstruction.^27^ But these complications can be controlled by symptomatic treatment like anti-infection treatment, semi-liquid nutrient intake, and replacement of PEG tube. A multivariate analysis has shown that the main risk factors affecting the one-year survival of PEG patients are summarized as hypoproteinemia, reduced blood lymphocyte count and malignancy-related complications.^28^ Interestingly, a former survey showed that ‘too old to suffer from an operation’ and leakage were common reasons for patients to refuse PEG.^15^ However, a study containing 201 patients (age >65) has indicated that old age is not a contraindication of PEG.^29^ Besides, leakage was not observed in our study. The reasons that patients refuse to use PEG are associated with the cultural values and education level of patients,^15^ therefore, it is necessary to state the advantage of PEG for patients in detail.

### Limitations of the study

Only 64 patients were included in this study, and the sample size was small. Therefore, in consideration of the small sample size, the clinical significance of this finding needs to be further confirmed.

## CONCLUSION

Our study indicates that nutrition support by PEG can significantly improve the nutrition status of critically ill patients. Additionally, cautious screening of cases, right selection of surgery timing and careful post-operative care are important for the success of surgery. It is still needed to develop ways to decide the risk/benefit ratio in the individual patient in order to optimize the timing and route of nutritional support.
